# Home energy management (HEM) database: A list with coded attributes of 308 devices commercially available in the US

**DOI:** 10.1016/j.dib.2017.10.067

**Published:** 2017-11-03

**Authors:** Marco Pritoni, Rebecca Ford, Beth Karlin, Angela Sanguinetti

**Affiliations:** a Lawrence Berkeley National Laboratory, Berkeley, CA, USA; b University of Oxford, Oxford, UK; c See Change Institute, Los Angeles, CA, USA; d University of California, Davis, Davis, CA, USA

**Keywords:** Home energy management, Energy efficiency, Smart home, Home automation, Internet of things

## Abstract

Policymakers worldwide are currently discussing whether to include home energy management (HEM) products in their portfolio of technologies to reduce carbon emissions and improve grid reliability. However, very little data is available about these products. Here we present the results of an extensive review including 308 HEM products available on the US market in 2015–2016. We gathered these data from publicly available sources such as vendor websites, online marketplaces and other vendor documents. A coding guide was developed iteratively during the data collection and utilized to classify the devices. Each product was coded based on 96 distinct attributes, grouped into 11 categories: Identifying information, Product components, Hardware, Communication, Software, Information - feedback, Information - feedforward, Control, Utility interaction, Additional benefits and Usability. The codes describe product features and functionalities, user interaction and interoperability with other devices. A mix of binary attributes and more descriptive codes allow to sort and group data without losing important qualitative information. The information is stored in a large spreadsheet included with this article, along with an explanatory coding guide. This dataset is analyzed and described in a research article entitled “Categories and functionality of smart home technology for energy management” (Ford et al., 2017) [1].

**Specifications Table**

**Table:** 

Subject area	*Energy efficiency*
More specific subject area	*Energy Management technology and Internet of Things*
Type of data	*Tables (spreadsheet file) and graphs*
How data was acquired	*Review of publicly available information gathered, categorized and summarized by coders*
Data format	*Raw and classified*
Experimental factors	–
Experimental features	*Codes were developed to systematically collect detailed data about each HEM product. Coders collected all the information in tabular format.*
Data source location	*Data collected on the Internet. Excluded products that were not commercialized in the US.*
Data accessibility	*Included with this article*

**Value of the data**

• This is the largest public dataset (more than 300 devices coded over 96 attributes) describing features of commercially available HEM products to date (2017).
• It can be used by researchers to identify product features and their potential to save energy and peak demand (Ford et al., 2016).
• It can inform policymakers who are evaluating whether to support HEM products in energy efficiency programs.
• It can inform the design of new devices through comparison with existing products.
• It can foster interdisciplinary research in energy efficiency, information technology and cyber-physical systems.

## 1 Data

The data presented in this article is related to the research article by Ford et al. [1], which reviews 308 home energy management (HEM) products available on the US market in 2015–2016, classify them using content analysis and explore their potential to deliver benefits to users and the grid. The raw data used for the analysis in [1] is available in this article in the form of a spreadsheet (Supplementary material). An excerpt of the data is shown in Table 1.

**Table 1:** Small excerpt of the data available in the Supplementary material.

**Developer/make**	**Model**	**Smart thermostat**	**Sensors - Temperature**	**Sensors - Humidity**	**Detects - Occupancy**	**Additional Hub/gateway required**	**Hub/gateway required - specify**
Allure Energy	Eversense	1	1	0	0	0	NA
Carrier	COR	1	1	1	0	0	NA
Centralite	Pearl Thermostat	1	1	1	0	1	ZigBee HUB
Control4	Control4 Wireless Thermostat by Aprilaire	1	1	1	1	1	Control4 Hub
ecobee	ecobee3	1	1	1	1	0	NA
Emerson	Sensi Wi-Fi Thermostat	1	1	0	0	0	NA
First Alert	First Alert Onelink Thermostat	1	1	0	0	0	NA
Honeywell	Lyric	1	1	1	1	0	NA
Honeywell	Wi-Fi Smart Thermostat RTH9580WF	1	1	1	0	0	NA
Insteon	Smart Thermostats	1	1	1	0	1	Insteon hub
Lowes	Iris Smart Thermostat	1	1	0	0	1	Lowes Hub
LUX	GEO 7-Day Wi-Fi Programmable Thermostat in White	1	1	0	0	0	NA
Nest	Nest Learning Thermostat	1	1	1	1	0	NA
Radio Thermostat of America	Thermostat CT 80 + WiFi module	1	1	1	0	0	NA
VENSTAR	ColorTouch	1	1	1	0	0	NA
Zen	Thermostat	1	1	0	0	1	ZigBee HUB

## 2 Experimental design, materials and methods

**Fig. 1:**
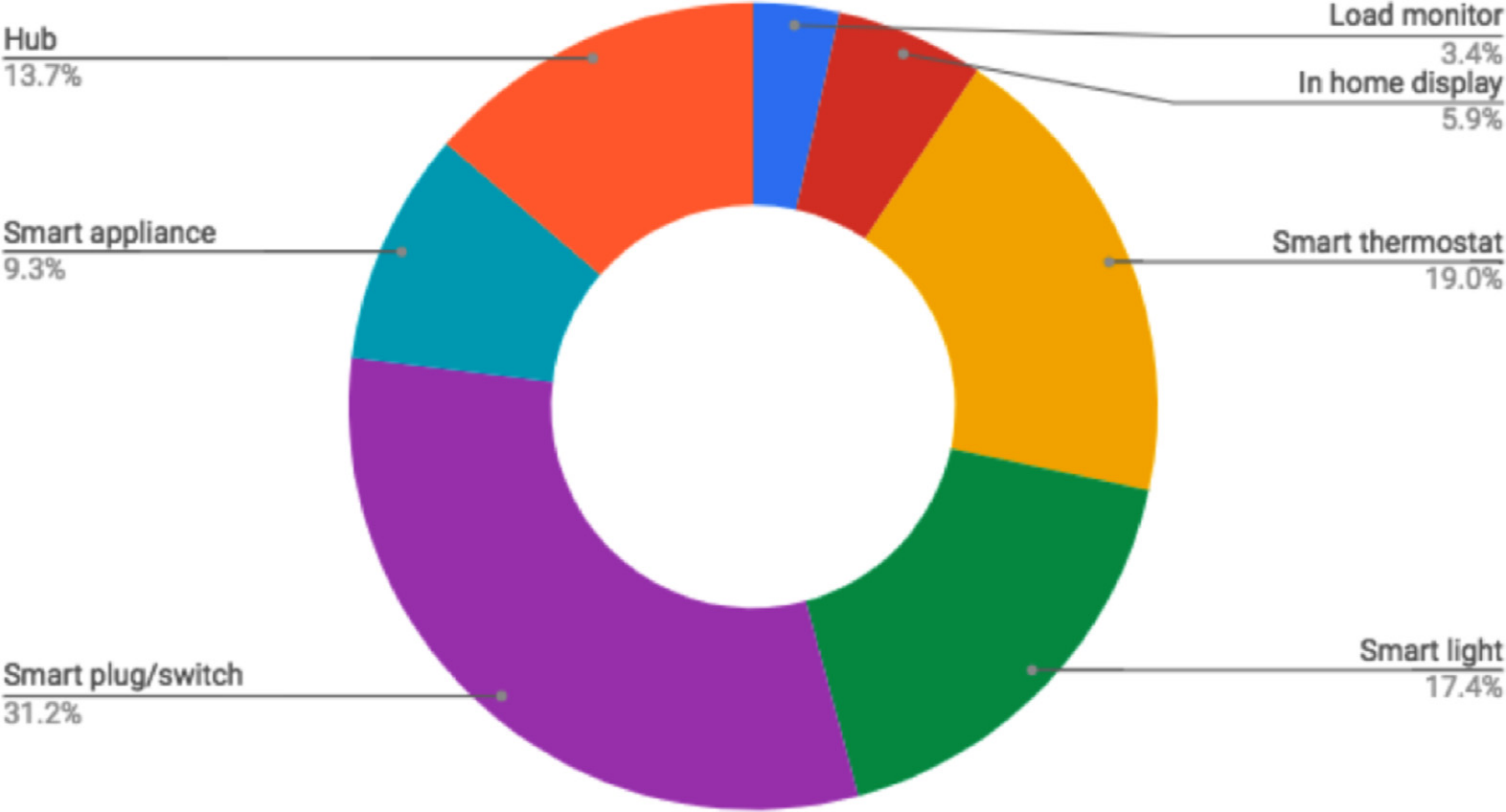
Breakdown of products reviewed by product category (see Table 2 in Ford et al. [1]).

The authors selected the 308 devices presented here from an initial list of more than 550 technologies identified from previous studies [3], [4], [5] and new sources [2]. The inclusion criteria and coding methodology are detailed in [1]. HEM devices reviewed include: load monitors, in home displays, smart thermostat, smart lights, smart plugs/switches, smart appliances, hubs. A breakdown of product types analyzed is presented in Fig. 1.

Codes were developed to collect and classify detailed data about each HEM product. A brief description each code category is presented in (Table 2, [1]). In Table 2.1–2.11 in the spreadsheet in Supplementary material (Coding Guide tab) we show the full coding guide developed, which describes the codes used in the database (see HEMS database tab).
