# Supplementary material for: Home energy management (HEM) database: A list with coded attributes of 308 devices commercially available in the US
Source: Data Brief. 2017 Nov 3;16:71–4. doi: 10.1016/j.dib.2017.10.067 (PMC5709341; doi:10.1016/j.dib.2017.10.067)
Supplement: Supplementary file 1 — Transparency document [file mmc1.doc]

Dear Editor and Reviewers,

Regarding our submitted paper (DIB-D-17-00642), we thank the reviewers for their comments which we addressed in the revised manuscript.

We hope you will find our revised study suitable for publication in *Data in Brief*.

The authors acknowledge that the submission declaration has been complied with (this paper has not been published previously, it is not under consideration for publication elsewhere, an its publication is approved by all authors), that they have no conflict of interest and that they have obtained all copyright permissions.

Sincerely,

Marco Pritoni, PhD

Lawrence Berkeley National Laboratory

1 Cyclotron Rd (Office 90-2066)

Berkeley, CA 94720

Phone: + 1 530 220 4394

E-mail: marco.pritoni@gmail.com
